# A transcytotic transport mechanism across the tympanic membrane

**DOI:** 10.1038/s41598-021-04748-w

**Published:** 2022-01-19

**Authors:** Arwa Kurabi, Kwang Pak, Eduardo Chavez, Jennifer Doan, Allen F. Ryan

**Affiliations:** 1 Department of Surgery/Otolaryngology, University of California, 9500 Gilman Drive, La Jolla, CA 92093-0666 USA; 2 Department of Neurosciences, University of California, San Diego, USA; 3 Department of Biology, University of California, San Diego, USA; 4 San Diego VA Healthcare System, La Jolla, CA USA

**Keywords:** Biochemistry, Molecular biology, Diseases, Medical research, Molecular medicine

## Abstract

Drug treatments for middle ear diseases are currently delivered systemically, or locally after opening the impermeable tympanic membrane (TM). We previously used bacteriophage display to discover novel peptides that are actively transported across the intact TM, with a variety of transport rates. Peptide structures were analyzed for evidence regarding the mechanism for this unexpected transport, which was then tested by the application of chemical inhibitors. Primary sequences indicated that trans-TM peptides share one of two amino acid motifs. Secondary structures revealed that linear configurations associate with higher transport rates than coiled structures. Tertiary analysis indicated that the shared sequence motifs are prominently displayed at the free ends of rapidly transported peptide phage. The shared motifs were evaluated for similarity to known motifs. The highest probability matches were for protein motifs involved in transmembrane transport and exosomes. Overall, structural findings suggest that the shared motifs represent binding sequences. They also implicate transcytosis, a polarized cell transport mechanism consisting of endocytosis, transcellular transport, and exocytosis. Inhibitor studies indicated that macropinocytosis, retrograde transport through Golgi and exocytosis participate in transport across the TM, consistent with transcytosis. This process can be harnessed to noninvasively deliver therapeutics to the middle ear.

## Introduction

Middle ear (ME) diseases include otitis media (OM), which in developed countries affects up to 90% of children^1,2^ and is a chronic/recurrent condition in 15–20%^3,4^. In many developing countries, serious OM is more common^5^. Undertreated OM is associated with an estimated 28,000 annual deaths^6^. OM is also estimated to cause one-half of the world’s burden of serious hearing loss, approximately 225 million cases, making it the world’s leading cause of deafness^6,7^. Cholesteatoma, in which epidermal cells invade the ME and exhibit aggressive and destructive growth, can erode middle and inner ear structures leading to hearing loss or even complete deafness^8^.

OM is primarily treated with systemic antibiotics or in more refractory cases, installation of pressure equalization tubes into a surgical opening in the tympanic membrane (TM)^9^. While these therapies are often effective, they have potential side effects. The use of systemic antibiotics for such a common disease contributes to the development of antibiotic resistant bacterial strains throughout the body^10^. Due to effects on the gut microbiome, they can also produce gastric distress^11^ which can be serious in infants^12^. Ventilation tubes can result in TM scarring or tympanosclerosis.

Cholesteatomas must be surgically removed, but complete resection is frequently impossible in the complex ME and mastoid space, leading to high-risk of recurrence^13^. Drug treatments to inhibit cholesteatoma growth would be valuable, but tissue growth inhibitors delivered systemically have significant side effects.

The local delivery of pharmacotherapy to the ME would be a very useful alternative to systemic drugs. However, the TM is an impermeable structure. Surgically breaching the TM is currently required for local drug delivery to the ME. Previous experimental studies have used tissue permeants^14^ or magnetically driven nanoparticles to transport drugs across the TM^15^. However, no methods have yet been demonstrated to be effective or safe for human use.

Many epithelial barriers have mechanisms for the active transport of macromolecules across their cells^16,17^. To determine whether this might also be true for the TM, we used the technique of phage display to search for peptides that would be able to cross the membrane. This method, introduced in the 1980s^18^, has been used in numerous biological systems to identify peptides and other molecules with specific properties, including drug delivery. The method employs very large combinatorial libraries of bacteriophage, each expressing a random peptide on its surface. The libraries are typically screened through multiple rounds for specific characteristics^17^. A primary advantage of phage display is that no prior knowledge of an interacting ligand is required. Rather, the desired biological activity is used to identify a peptide sequence that will interact with the desired target process. Since a large number of targeting peptides have been identified using phage display^17^, we chose this method for application to the TM. We reasoned that if repeated screening of the library for trans-TM transport led to collapse of a naïve library expressing a large number of peptides to a small number of sequences, this would potentially identify clones that can target the TM and enter the ME.

We screened a 12-mer phage library (PhD-12™, New England Biolabs), as described previously^19^. We employed sequential screening for peptides that bound to, internalized into and finally penetrated the TM. Two rounds of screening resulted in nine peptides (TMT 1–5 and BPT 1–4) that crossed the TM far more efficiently than phage without a peptide (termed wild-type phage), although a range of transport efficiencies was observed. Peptide phage movement across the TM was oxygen and temperature dependent, suggesting active transport^19^, and peptides without phage were also transported.

These studies identified peptides with the capacity to actively cross the intact TM carrying large (1 μm) cargo. The peptides are not similar to known cell- or tissue-penetrating peptides^19^. The discovered mechanism of trans-TM transport offers the opportunity to efficiently deliver large-molecular weight drugs, gene therapy vectors and other cargo to treat ME disease.

The mechanism by which trans-TM transport occurs is unclear. There are two primary mechanisms of transport across cellular barriers^20^. One is paracellular transport in which intercellular tight junctions are temporarily loosened allowing substances to pass between cells. The other is transcellular transport mediated by specific transmembrane transporters or by cross-cellular transport of the contents of vesicles.

The purpose of this investigation was to compare the structure of trans-TM peptides with the greatest transport level, to identify possible structural features that support rapid transport and provide clues to the transport mechanism, and to test implied processes.

## Results

### Motif analysis

**Figure 1:**
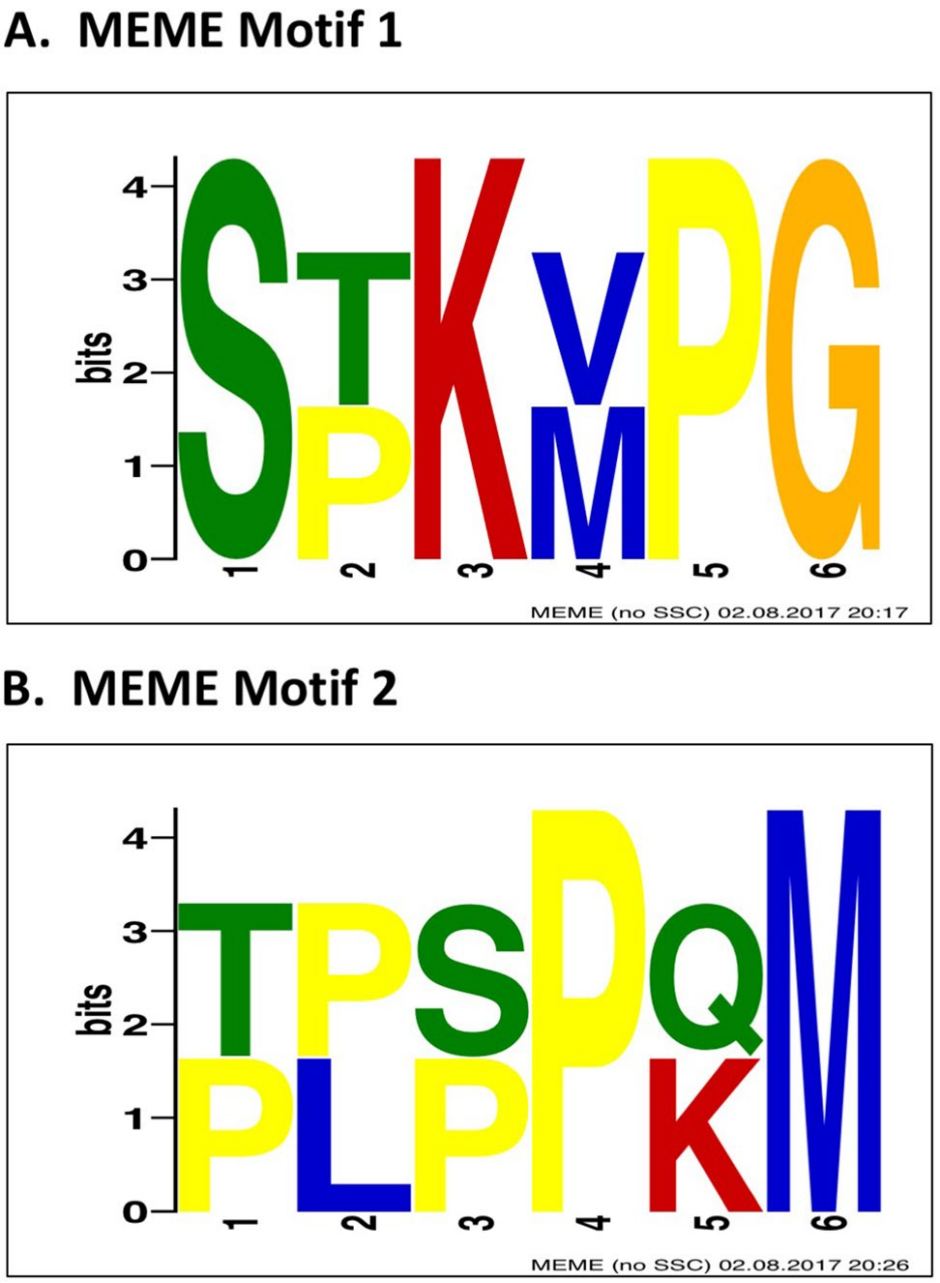
The motifs identified by MEME analysis in the nine peptides for which transport data were obtained (**A**. Motif 1), and analysis limited to the five peptides with the highest transport rates (**B**. Motif 2). These motifs highlight the importance of a central lysine (K) or proline (P) in the peptides.

MEME analysis^21^ was used to expand upon two motifs that we previously identified^19^ in trans-TM peptides (ST^K^/_R_T and PxxP). When all nine peptides for which transport rates had been determined were analyzed, a six amino acid motif related to ST^K^/_R_T was detected (Motif 1, Fig. 1A). When the five highest transporting peptides were analyzed, a six amino acid motif related to PxxP was detected (Motif 2, Fig. 1B).

### 12-mer peptides and transport rates

The sequences and ME recoveries of nine 12-mer peptides were documented in a prior publication^19^. Based on their ME recovery after a 1-h incubation on the TM, the peptides and rates were divided into transport categories, high, medium high, and low (Table 1).

**Table 1:** Sequences and ME recovery of trans-TM 12-mer peptide phage identified by two screening methods (data from Kurabi et al.^19^).  Each purified phage was applied to the TM of previously infected rats for one hour (n = 6), and the ME contents then tittered and the number of phage particles recovered were averaged (mean of n = 6). Phage recovery was grouped into transport categories, which were then used to generate Fig. 1.

Transport category	Name	Sequence	Mean recovery from ME (PFUs)	Standard error
High	BPT-3	THPSTKVPGTPA	13,050	6700
	TMT-3	SADSTKTTHLTL	12,030	4500
	BPT-4	TFNPPPPQMPST	9040	3200
Med/High	TMT-4	DVGAGRWFSDNG	5430	1100
	TMT-2	TLSPKMPGGGYW	3210	1250
	TMT-5	SDDSRPIAQFAI	3017	960
Low	TMT-1	SPPGKFLESLRS	610	350
	BPT-2	ALWPPNLHAWVP	210	65
	BPT-1	TPMVERNYNAAD	95	42

### Secondary structural analysis

**Figure 2:**
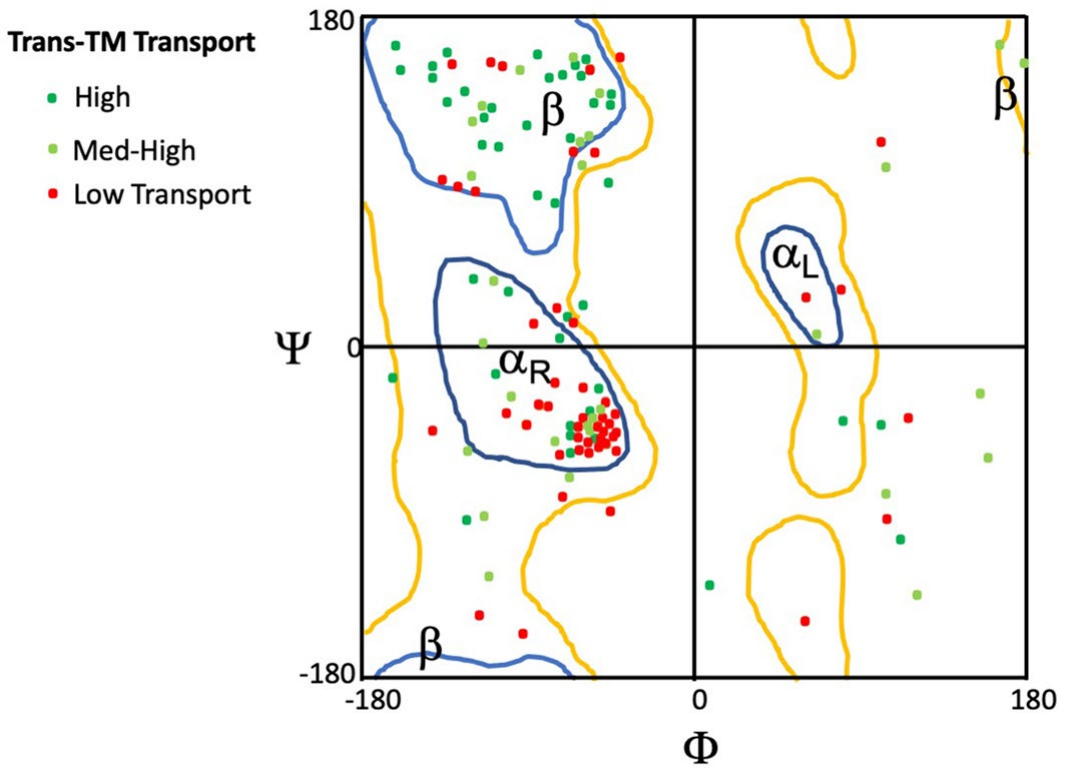
Ramachandran plot for 12-mer peptides expressed by trans-TM peptide phage with differing rates of transport across the membrane (Table 1). The plot predicts the orientation of the amino acids in a peptide backbone based on their side chains. Orientations consistent with β-string (β), right-handed α-coil (α_R_) and left-handed α-coil (α_L_) structures are outlined. When peptides from different trans-TM transport categories are plotted, the peptides with the highest rates (green) show a strong tendency to fall into the area consistent with β-string formation. The amino acids of those with the lowest rates (red) cluster in the area most consistent with right-handed α-coil structure.

The 3-D structures of the nine peptides predicted by PEP-FOLD were subjected to Ramachandran analysis of amino acid dihedral backbone angles to predict peptide structure. The individual plots were then combined in an overlay, and the amino acids of each peptide were color-coded to reflect their transport rate categories as presented in Table 1. The results of this analysis are presented in the Ramachandran plot of Fig. 2. As can be seen in the figure, the orientation of amino acids in the peptides exhibiting the highest levels of trans-TM transport tended to fall into the + 180 degree ψ and -180 degree φ quadrant of the plot, consistent with linear, β-chain formation. Those exhibiting the lowest transport across the TM were clustered in the -180 degree ψ and -180 degree φ quadrant, consistent with a propensity for forming a right-handed, coiled α-helical structure.

**Figure 3:**
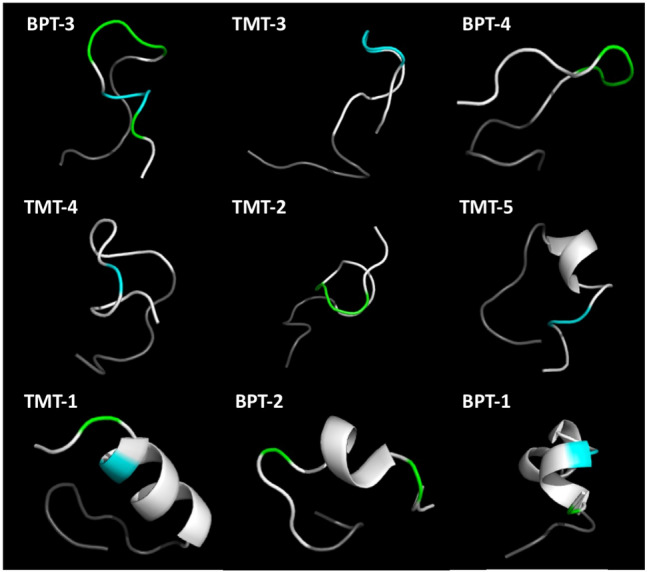
Secondary structure of trans-TM peptides, in order of decreasing transport rate from top left to bottom right. Coiled-coil structure is clearly associated with the lowest transport rates.  The structures are oriented with the terminal six amino acids of the M13 phage pIII protein, colored gray, on the lower left.   The amino acids of Motif 1 are indicated in blue, while those of Motif 2 are indicated in green.

The secondary structures predicted by PEPFOLD are illustrated in Fig. 3. The structures are arranged in order of ME recovery after 1 h on the TM (see Table 1). The motifs identified in the MEME analysis are indicated in blue (Motif 1, ST^K^/_R_T) and green (Motif 2, PxxP). As indicated by the Ramachandran analysis, coiled-coiled peptide structure is visibly associated with the lowest trans-TM transport rates.

### Tertiary structural analysis

**Figure 4:**
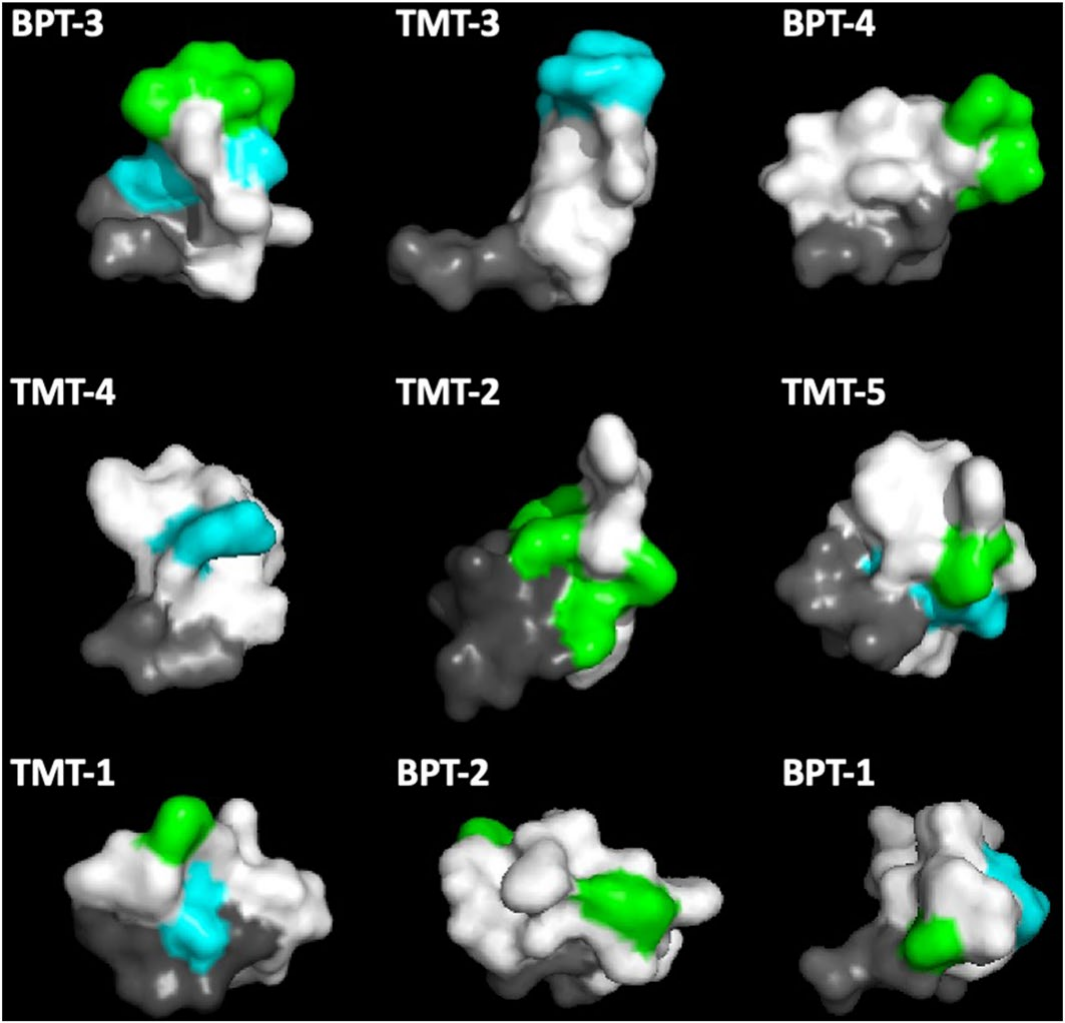
3-D structures for the nine peptides predicted by PEPFOLD, again arranged in order of transport rate (Table 1) from top left to lower right. The structures are oriented and colored same as in Fig 3.  Peptide structures with the highest transport rates display motifs that are exposed at the free end of the peptide. Those with lower transport rates exhibit fewer motif amino acids on their surfaces, and those present tend not to be situated at the pIII free peptide end.

Figure 4 illustrates the surface structures of the nine peptides as generated by the PEPFOLD 3-D prediction suite. The structures are again arranged in order of ME recovery after 1 h on the TM. In the figure, the N-terminal pIII amino acids are indicated in gray, with the 12-mer peptides oriented relative to them. The amino acids of the ST^K^/_R_T motif are colored blue, while those of the PxxP motif are encoded green. It is clear that for the peptides in the highest transport rate category, more motif amino acids are present in the sequence, and a motif is more prominently displayed at the end of the predicted structure. In these high-transport peptide structures, the motifs would presumably be highly available for binding to sites on the TM. The remaining peptides exhibit increasingly lower numbers of motif amino acids, and structures in which other amino acids could interfere with ready access of these incomplete motifs to sites on the TM.

The two MEME motifs were subjected to further analysis by the MAST application (Table 2), the highest sequence alignment for the motifs identified in the nine-peptide screen resulted in a match for *exoc1* which encodes exocyst complex component-1^22^. EXOC1 mediates docking of exocytic vesicles with fusion sites on the plasma membrane. The highest sequence alignment in the MAST output for the motifs identified by MEME analysis of the five highest transporting proteins was to KPNA1, which encodes importin subunit alpha 6. The importin complex is involved in transport of proteins across the nuclear membrane^23^, these findings implicate transcytosis a transcellular transport mechanism involving vesicles.

**Table 2:** MEME/MAST motif identification and scanning resulted in a number of prominent motifs identified in the top TM penetrating peptides. Pattern analysis using the peptides sequences from Table 2 reveled the two featured conscience motifs. The sequence logos are depicted in the first column and the top MAST output hits (E-value > 10) are presented.

Motif logo predicted by MEME	MAST output scan of possible genes
Motif 1	Exocyst complex component 1 (*exoc1*)
	Golgi associated secretory pathway kinase (fam20c)
	Sortilin 1 (*sorl1*)
	Tetraspanin-5 (*tspan5*)
Motif 2	Importin subunit alpha (*kpna6*)
	Zinc fingers homeobox protein 1 (No vertebrate homologue)
	Secretory Carrier Membrane Protein 5 (*scamp5*)
	GABA Type alpha 4 subunit (*gabra4*)

### Inhibitor studies

**Figure 5:**
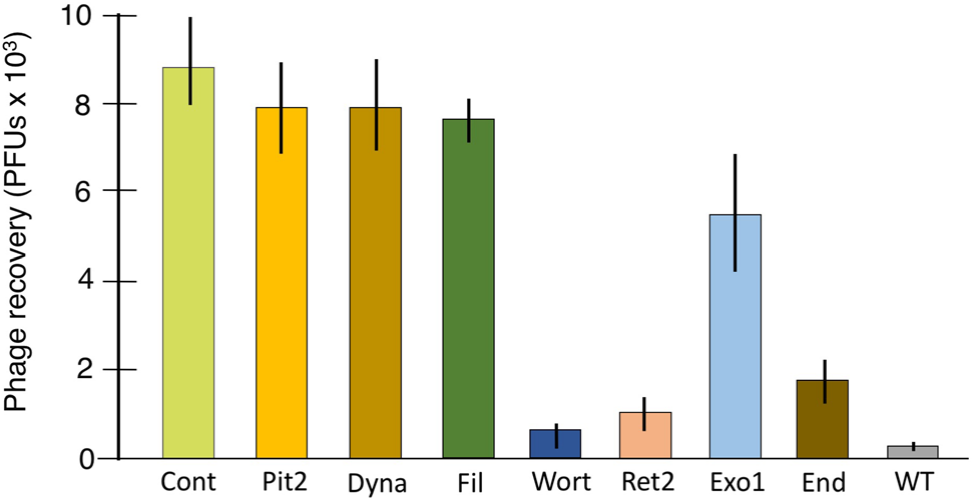
Effects of various inhibitors on trans-TM transport of phage expressing peptide TMT-3. Inhibitors of clathrin-dependent and most clathrin-independent mechanisms of endocytosis had no effect on trans-TM transport. Inhibitors of macropinocytosis (wortmannin), retrograde transport into the Golgi network (Retro-2) and exocytosis (Exo-1 and Endosidin-2) significantly reduced transport (n = 6 ears).

Phage bearing the trans-TM peptide TMT-3 was used to explore the role in TM transport of various processes potentially involved in transcytosis, which consists of endocytosis and transcellular transport followed by exocytosis. Figure 5 illustrates the results of inhibitor studies.

Endocytosis is the initial and required step in transcytosis. There are many forms of endocytosis. The best characterized of these is clathrin-mediated endocytosis, which is often associated with receptor internalization. In response to receptor activation and other signals, clathrin forms lattices on the interior cell membrane surface that induce the formation of membrane pits^24^. We evaluated two inhibitors of clathrin-mediated endocytosis. Chlorpromazine inhibits clathrin-mediated endocytosis by preventing the assembly and disassembly of clathrin lattices on cell surfaces and on endosomes^25^. Pitstop-2 inhibits of clathrin activity through interactions with the clathrin molecule at multiple sites^26^, although it also inhibits some forms of clathrin-independent endocytosis^27^. Neither Pitstop-2, as illustrated in Fig. 5, nor chlorpromazine had any effect on trans-TM phage transport.

Most forms of clathrin-independent endocytosis depend upon caveolae, small invaginations in membrane lipid rafts. We evaluated several inhibitors of caveolar endocytosis, including genistein, nocodazole, N-ethymaleimide, carrageenan and Filipin, all of which disrupt lipid rafts^28^. As illustrated for Filipin in Fig. 5, there was no effect on trans-TM transport for any of these inhibitors.

Both caveolae and clathrin-mediated membrane pits are excised into endosomes by dynamin, which cleaves pits from the membrane. We tested Dynasore, a dynamin inhibitor that can also disrupt actin filaments^29^. As shown in Fig. 5, Dynasore had no effect on trans-TM phage transport.

A clathrin-independent form of endocytosis that does not involve caveolae or dynamin is macropinocytosis, a clathrin-independent and nonspecific endocytotic mechanism used by many viral pathogens for cell entry. Macropinocytosis operates via rapid polymerization of actin-rich structures that rise up from the cell surface before collapsing back down into a macropinosome, a process that is dependent upon PI3 kinase (PI3K)^30^. Treatment of the TM with the PI3K inhibitor wortmannin^31^ reduced trans-TM transport by approximately 90% (Fig. 5).

Once cargo is internalized into endocytes, transcellular transport can be continued via early endosome fusion into late endosomes^32^ or into common recycling endosomes^33^, or by retrograde transport into the Golgi apparatus. Bafilomycin, an inhibitor of transport from early to late or recycling endosomes^34^, had no effect on trans-TM transport (Fig. 5). In contrast, Retro-2, which inhibits retrograde transport from endosomes into the Golgi network^35^, reduced trans-TM transport by approximately 90% (Fig. 5).

The final stage of transcytosis is transport into exosomes, followed by exocytosis. Exo-1, an inhibitor of transport from Golgi to the endoplasmic reticulum, a step in the formation of Golgi-derived exosomes^36^, reduced trans-TM transport by 40% (Fig. 5). Endosidin-2 is an inhibitor of EXO70, a subunit of the exocyst complex involved in exocytosis^36^, reduced transport by ~ 80% (Fig. 5).

## Discussion

Structural analysis of trans-TM peptides offered important information regarding characteristics related to trans-TM transport rate. Figure 2 clearly suggests that a β-chain structure is associated with the highest observed transport rates. 12-mer peptides are too short to form β-sheets, so β-chain structure is likely associated with a relatively flexible and unstructured linear configuration. This could allow freer interaction with protein substrates that might mediate active trans-TM transport.

Artificial β-chains, as in the short phage display peptides studied here, can be subject to aggregation and insolubility due to the exposed hydrophobic side of the chain. Natural β-chains are protected from this by various structural motifs, including adjacent chains that mediate the formation of less reactive sheet structures, abrupt angles such as those produced by prolines, and the inclusion of a hydrophilic amino acid in the middle of the β-chain^37^. For example, the aggregation of β-amyloid β-chain protein fragments, which contribute to Alzheimer’s disease, can be reversed by introducing a lysine into the middle of the fragment^38^. In this regard, it is interesting to note that the trans-TM peptides detected in our studies are characterized by central lysines and/or prolines (Fig. 1). It is possible that these residues preserve peptide solubility and flexibility. The perseverance of the conformational freedom on these peptides would be important, as it could facilitate multisite targets and interactions with different receptors or cell types.

The predicted 3-D surface structure of the peptides indicates that the two motifs present in the peptides are critical to transport across the TM. The three peptides with the highest rate of trans-TM transport not only exhibit the most complete forms of these motifs, they also present the motifs at the free ends of their structures, where they would have ready access to any potential binding features on the surface of the TM. Peptides with successively lower transport rates are characterized by partial sequences of these motifs, and locations closer to the phage pIII protein, where they could be blocked by other amino acids.

It is important to note that the exact confirmation of the peptides presented at the N-terminal tail of pIII protein maybe difficult. Our peptides are used on the tympanic membrane in solution, at the temperature and pH at which they were modeled using PEP-FOLD. Moreover, PEP-FOLD is widely used in drug design^39^ and de novo peptide structure perdition from primary sequence. Comparison of PEP-FOLD with other algorithms has found that its performance is generally superior, especially for peptides in the 9–15 amino acid range, in predicting the structure of peptides for which the crystal structure have been determined^40^. These peptides models are mainly used to enable greater understanding and possible clues of their unique TM transport function.

**Figure 6:**
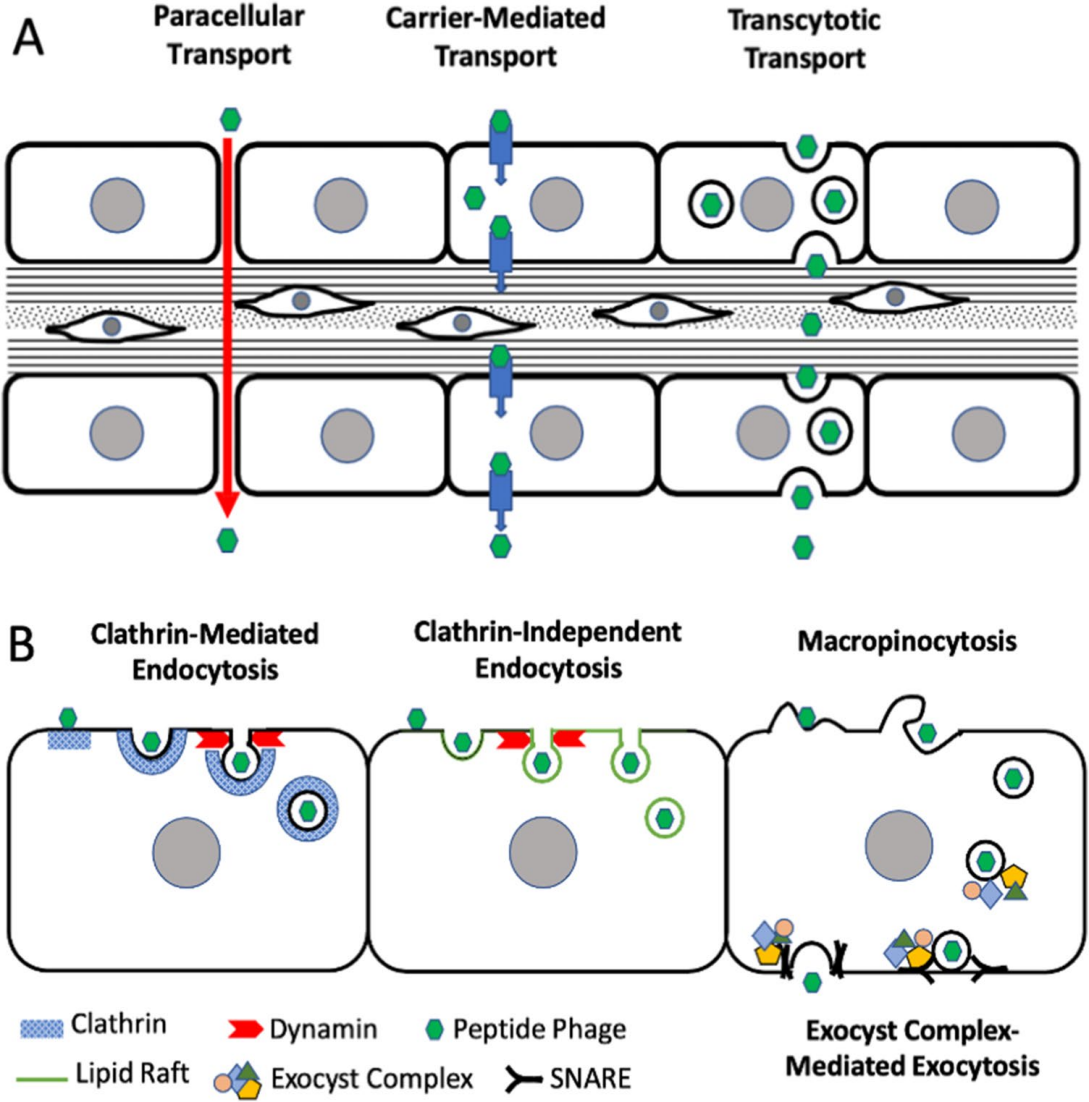
(**A**) Possible mechanisms of peptide phage transport across the TM. Paracellular transport is not active and unlikely since trans-TM transport is oxygen- and temperature-sensitive. Carrier-mediated transport is limited to relatively small molecules. Transcytosis can move large cargo across polarized epithelia in either direction, and was thus investigated. (**B**) Potential mechanisms involved in transcytosis. Inhibitors of clathrin or dynamin did not block trans-TM transport, ruling out clathrin-mediated endocytosis. Several forms of clathrin-independent endocytosis including caveolar and caveolin-independent endocytosis occur in cholesterol-rich lipid rafts, but cholesterol depletion did not affect trans-TM transport. Inhibition of macropinocytosis, capable of endocytosing large (> 500 nm) particles including viruses, virtually eliminated trans-TM transport, as did inhibition of exocytosis mediated by the exocyst complex, implicating participation of both processes in trans-TM transport.

As noted above, transport across the TM could most probably be accomplished by one of three basic mechanisms (Fig. 6). Paracellular transport would seem a likely mechanism by which to transport large cargo such as bacteriophage. The paracellular pathway of transport functions in the gut epithelium and is important for the absorption of some nutrients and drugs in the gastrointestinal tract^41^. The paracellular mechanism also has the attraction that it could function for TM cells of either polarity. However, paracellular transport is not an active process, but rather involves passive movement along a concentration gradient once gaps are introduced in tight junctions^41^. This is inconsistent with the transport of trans-TM peptides, which we have shown to be active^19^.

Transcellular transport across the TM could be mediated by one or more specific channels or molecular transporters to accomplish the movement of peptides and attached cargo across the membranes of TM cells. There are many transmembrane molecules that actively transport important molecules either into or out of cells (e.g. ^42^.). However, we know of no membrane transporters that move particles the size of bacteriophage through cell membranes, nor of channels that are large enough for bacteriophage entry.

Alternatively, transport could be by transcytosis^43,44^. Many polarized cells use the endocytic pathway to shuttle cargo between their distinct membrane domains, including apical and basal membranes. In transcytosis, cargo is internalized at the membrane on one side of a polarized cell by endocytosis. This can be the apical side of a cell, as in gut epithelial cell transport of nutrients, or the basal side of the cell as in endothelial cell delivery of IgA to the vessel lumen^45^. Cargo delivered first to early endosomes can be shuttled via a common set of late endosomes and then through basolateral early endosomes, for fusion to the plasma membrane of the opposite side of the cell^44^. Otherwise, the contents of early apical endosomes can be retrogradely transport into the Golgi complex for transport and packaging into transport endosomes that then fuse with the basolateral membrane^44^. Via these mechanisms transcytosis selectively moves material through cells and across tissue barriers; for example, from the luminal (apical) side to the underlying interstitium (basolateral) side of endothelial cells that line blood vessels, or the epithelium that lines the intestines, or in the case of IgA from the basolateral side of endothelial cells to the apical side^44,45^.

Even though this transport is mediated by endosomes, components of the exocytotic system, like the tethering exocyst complex and SNAREs, are required for and regulate this process^43^. If transcytosis exists in the TM, this mechanism could be harnessed by trans-TM peptides, binding to elements that are transported between the apical and basal plasma membrane domains of TM cells.

Supporting the possibility of transcytosis, motifs identified in our peptides were related to motifs in two molecules that are components of the exocyst and importin transport mechanisms across cell membranes. While the structure and interaction of these complexes are not yet known, it seems possible that the motifs that we identified in these molecules support binding to elements of the complex. Trans-TM peptides might similarly bind, and be carried through the cells of the TM.

The results of Pitstop-2, Dynasore, and Filipin treatment indicate that clathrin-dependent and most forms of clathrin-independent endocytosis are not involved in peptide-mediated trans-TM transport. However, inhibition by wortmannin, an inhibitor of PI3K, provides strong evidence supporting a critical role for macropinocytosis. Macropinocytosis is an important form of endocytosis responsible for uptake of compounds in fluid phase, for a number of cell types^46,47^. It occurs via actin-dependent reorganizations of the plasma membrane to form morphologically diverse vesicles that lack the coat structures typical of many endosomes and do not require dynamin for excision from the membrane. Macropinocytosis can be constitutive, but in some cells it requires activation by ligands such as growth factors^48,49^. It is thus possible that trans-TM peptides act as ligands to induce macropinocytosis.

Inhibition of trans-TM transport by Retro-2 indicates that retrograde transport of endosomes into the Golgi apparatus is also a critical step in the process of transcellular movement. A number of viruses and toxins have been found to utilize retrograde transport into the Golgi apparatus for cell entry^50^. Moreover, vaccinia and monkeypox viruses employ retrograde transport into Golgi for wrapping their virions with a double-membrane envelope that enables microtubular transport and exocytosis^51^. Trans-TM peptides may use similar mechanisms for transport across TM cells. In contrast, fusion of early endosomes into late or recycling endosomes does not appear to pay a role in transport across the TM.

Inhibition of transport from Golgi into the endoplasmic reticulum by a high dosage of Exo1 partially reduced trans-TM transport, suggesting that the production of exosomes may be complex. Exosomes can also be released from Golgi via anterograde transport from the trans-Golgi network^52^, which might explain the partial inhibition. However, the more robust inhibition of transport by Endosidin-2 indicates that the exocytotic system and exocytosis are required for transport through the TM.

Taken together, our peptide structural and inhibitor data strongly support transcytosis as the mechanism by which peptides mediate active transport of cargo across the TM. Moreover, this process appears to be initiated by presentation of specific amino acid motifs to the surface of the TM, where they may serve as ligands binding to specific targets and initiating micropinocytosis.

Important questions regarding trans-TM transport remain unanswered. Active transport across the TM is a novel and unsuspected process. In previous papers we have established that increasing peptide length can, for certain sequence additions, enhance the rate of trans-TM transport^53^. While bacteriophage M13 expresses five copies of each peptide fused to the pIII protein at one end of the M13 phage, we have also shown that a single copy fused to a DNA template for qPCR analysis is transported across the tympanic membrane at a similar rate^53^, suggesting that the number of peptides does not determine transport efficiency. We have only tested individual cargoes (bacteriophage or DNA), but the large size of M13 bacteriophage suggests that transport of large peptide-labeled packages of cargo would be possible. It is of note that we only tested peptides which are fused to cargo at the C-terminus, with the N-terminus free to recapitulate how the peptides are fused through the C-terminus to the pIII protein in the engineered NEB phage library. Ultimately the biological purpose normally served by macropinocytosis and transcytosis across the TM is unknown. Macropinocytosis is involved in the growth of cancer cells, which scavenge nutrients, including amino acids and proteins, from surrounding dying cells^54^. It can be speculated that epidermal cells on the surface of the TM, which proliferate to renew the TM surface^55^ similarly derive nutrients from the sloughed epidermal cells that are present on the external TM surface, and may also transport these nutrients to cells deeper within the TM. However this hypothesis is unsupported by evidence, and does not explain transport into the ME.

### Clinical application

The isolation of peptides capable of actively transporting large particles (M13 phage are approximately 1 μm in length) across the TM provides a potential targeting mechanism for the delivery of diverse therapies into the ME. This could include drugs, drug packages, gene therapy vectors or even bactericidal phage. A noninvasive delivery mechanism would enhance the practicality of local delivery of antibiotics. Antibiotic therapy for OM is employed primarily in children, where surgical means of delivery require general anesthesia to prevent damage to the delicate structures of the ossicular chain and inner ear. Local application of antimicrobials would eliminate exposure of bacteria throughout the body, reducing the risk of antibiotic resistant strains. Gastric distress would similarly be eliminated. The simplicity noninvasive local drug delivery would also be beneficial in those developing countries for which limited access to advanced therapies means that OM is undertreated. This is estimated to cause 28,000 deaths/year, and to be responsible for half of the world’s burden of serious hearing loss (~ 225 million cases)^56^. Drugs to reduce the cholesteatoma growth and recurrence could also be delivered across the intact TM, without the need for revision surgery. Gene therapy to treat genetically mediated ME disease, or to impart resistance to an acquired ME disease, is another potential application. Finally, antibiotic resistance has renewed interest in the potential for lytic bacteriophage in the treatment of bacterial infections^57^. Bacteriophage therapy has been applied successfully to resolve longstanding otitis externa due to antibiotic-resistant *Pseudomonas* infection^58^. Bacteriophage-based therapies have the distinct advantage over antibiotics that phage can evolve in-step with bacteria, reducing the risk that resistant pathogen strains can develop. Of course there are still some barriers to clinical therapy including understanding and validating the long-haul phage pharmacokinetics on the TM, ME and inner ear for example. Currently we do not know details regarding the phage’s or the peptides ability to remain active *in vivo*. Longer-term safety and efficacy of trans-TM peptides transport need to be evaluated locally and systemically. The foreign peptides may instigate an immune response. In addition, a practical method for safe long-term delivery are required since the low bioavailability represents a weak point that requires further investigation and optimization.

### Future directions

The TM protein targets that mediate trans-TM transport are unknown, but could provide important clues as to its function. Using trans-TM peptides as bait to capture TM proteins is one strategy that we plan to employ.

Major questions for potential therapeutic applications include whether peptides can transport therapeutic cargoes in sufficient quantities needs to be determined. Linkage of peptides to antibiotics, antibiotic packages, and gene therapy vectors will be required to address this issue.

Whether peptides have any negative effects on the ME or inner ear is also critical to therapeutic application. We have shown that direct exposure of the ME to bacteriophages bearing TMT 1–4 for up to 72 h does not produce ME inflammation or impact hearing thresholds^59^. More detailed safety studies will be needed to determine the potential for clinical potential of active trans-TM transport.

## Methods

### Animals

Experiments were performed using Sprague Dawley rats to National Institutes of Health standards for the Care and Use of animals in research, and were approved by the San Diego VA Healthcare System IACUC. Studies were carried out in compliance with the ARRIVE (Animal Research: Reporting in Vivo Experiments) guidelines. All experiments were performed in accordance with the relevant guidelines and regulations on veterinary best practices.

### Peptide motif analysis

We previously^19^ noted an ST(K/R)T motif in peptides TMT-3, TMT-4, TMT-5 and BPT-3. An additional motif containing prolines, PxxP, was complete in peptides TMT-2 and BPT-4. Portions of these motifs were present in the other trans-TM peptides. Multiple Em for Motif Elicitation (MEME)^21^ (web version 4.12.0), was used to expand the motif analysis and identify longer motifs suitable for protein database alignment. For MEME analysis, FASTA files were generated for all 9 phage peptides present in Table 1 following the guidelines of the MEME suite. In addition, a de novo motif search using only the top 5 peptides was performed. The software was run with a site distribution of any number of sequences and to search for motif lengths up to 12 amino acids.

### Transport rates and peptide structure

The 12-mer peptides identified in our prior study^19^ exhibited a range of transport rates. To explore the basis of transport efficiency, we structurally analyzed each peptide as fused to the terminal amino acids of the M13 pIII phage surface protein, relative to transport efficiency. PEP-FOLD software^60^ was employed to predict peptide 2-D and 3-D structures present on the free end of the pIII protein. 3-D structures were used to generate Ramachandran plots^61^. The plots visualize energetically allowed regions for the peptide backbone dihedral angles, ψ against φ, and predict backbone orientation and potential structural motifs of the peptides.

### MEME motif alignment to proteins

Motifs identified by MEME analysis were aligned to known proteins, to provide potential information regarding the trans-TM transport mechanism. For each MEME motif hit, the sequence was input to MAST (Motif Alignment and Search Tool) software to search the Ensembl Ab initio protein sequence database and identify proteins in which motifs hits were present. Alignments were considered hits at *P* value < 0.0001 and E-value less than 10. UniprotKB was used to identify the known or predicted functions of top hits.

### Infection of the ME

Rats were anesthetized with rodent cocktail (2.0 mg/kg xylazine and 40.0 mg/kg ketamine i.m.). The ME bulla was exposed ventrally from a midline neck incision, a small opening made with a 20 g needle, and 5 × 10^4^ PFUs of nontypeable *Haemophilus influenzae* (NTHi) was injected in 50 μl PBS.

### Inhibitor studies

The MEs of rats were infected with NTHi as above. 48 h later, the external ears of six anesthetized rats were injected with 50 µl of saline containing 10^10^ phage expressing TM-3 peptide and up to four inhibitors. Inhibitors were applied at 5–10 times the published IC50 (see Table 1). For any combination of that affected trans-TM transport, Inhibitors were then tested individually to identify the active agent(s). Inhibitors included bafilomycin A (2 µM), brefeldin A (1 uM), carrageenan (200 mg/ml), chlorpromazine (300 µM), Dynasore (500 µM), Endosidin-2 (1 mM), Exo-1 (200 µM), Filipin (50 µg/ml), genistein (100 µM), N-ethylmaleimide (50 µM), nocodazole (300 µM), PitStop-2 (100 µM), Retro-2 (100 µM) and wortmannin (100 µM). Six additional rat external ears were injected with 10^9^ TMT-3 bacteriophage without an inhibitor, as positive controls. Six external ears were injected with 10^9^ wild-type (WT) phage without a peptide, as negative controls for phage contamination. After one hour, the external ears were extensive rinsed to remove all phage, the MEs were opened and the contents titered to quantify bacteriophage entry^59^. Six replicates were averaged to get the mean and The standard error was calculated by dividing the standard deviation by the sample size's square root (n = 6).
